# Supplementary material for: RBD-Protein/Peptide Vaccine UB-612 Elicits Mucosal and Fc-Mediated Antibody Responses against SARS-CoV-2 in Cynomolgus Macaques
Source: Vaccines (Basel). 2023 Dec 29;12(1):40. doi: 10.3390/vaccines12010040 (PMC10818657; doi:10.3390/vaccines12010040)
Supplement: Supplementary file 1 [file vaccines-12-00040-s001.zip › vaccines-2757163-supplementary.pdf]

Supplemental Figure S1. Antibody-dependent monocyte phagocytosis (ADMP) in UB-612 immunized macaques

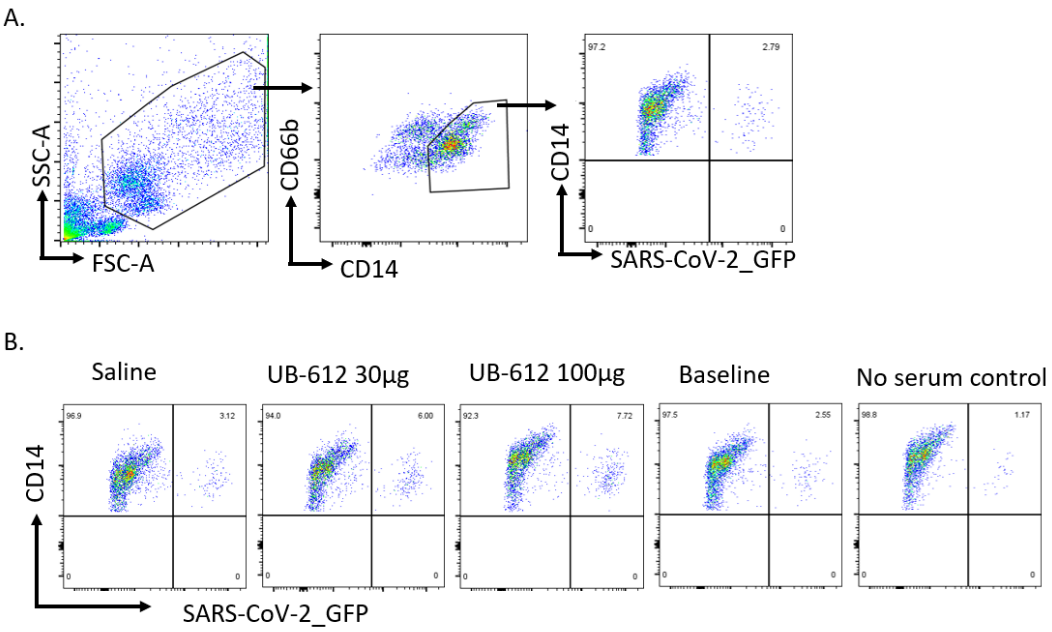

Supplemental Figure S2. ADNKA (A)

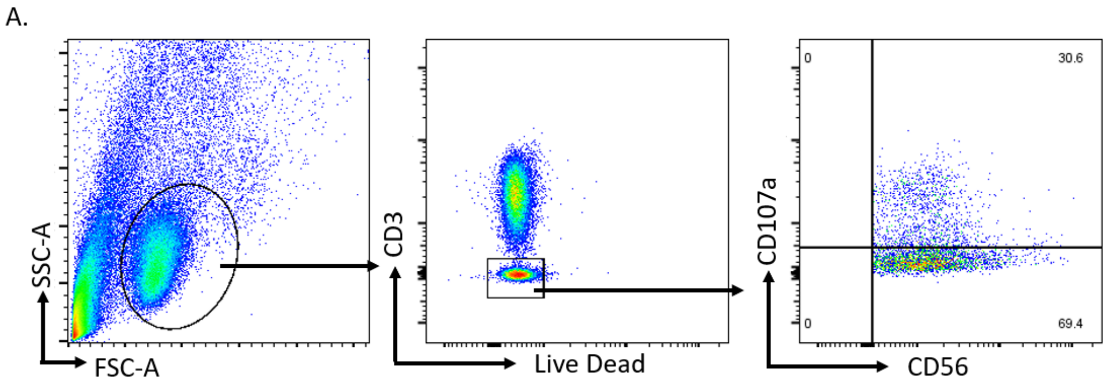

Supplemental Figure S2. ADNKA (B)

B.

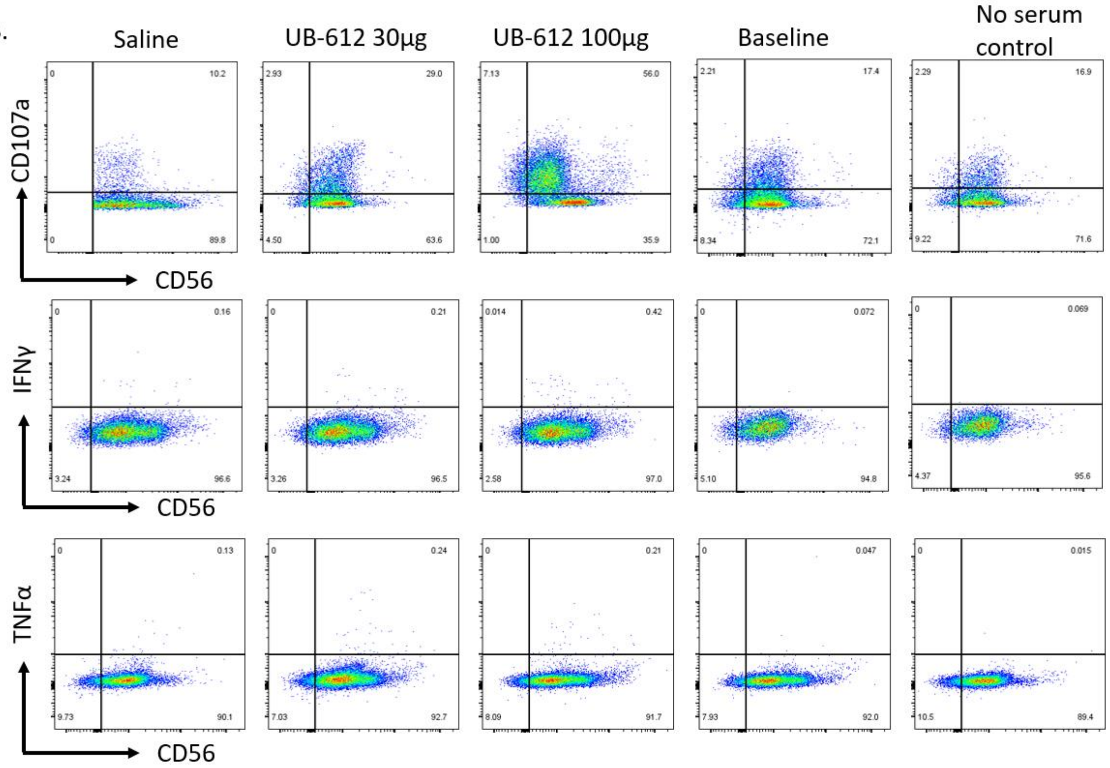

Saline
UB-612 30µg
UB-612 100µg
Baseline
No serum control
CD107a
CD56
IFNγ
CD56
TNFα
CD56
